# Antioxidant properties of *Moringa oleifera* Lam. hay and its effect on the reproductive capacity of rams

**DOI:** 10.1590/1984-3143-AR2025-0078

**Published:** 2026-07-27

**Authors:** Michele Pereira da Silva, Hymerson Costa Azevedo, Evandro Neves Muniz, Rafael Dantas dos Santos, Ana Mara de Oliveira e Silva, Larissa de Oliveira Queiroz, Iranildo Soares Bispo, Matheus Batista de Oliveira, Julio Constantino Jerí Molina, Gladston Rafael de Arruda Santos

**Affiliations:** 1 Universidade Federal de Sergipe – UFS, Departamento de Zootecnia, São Cristóvão, SE, Brasil; 2 Universidade Federal de Sergipe – UFS, Programa de Pós-graduação em Agricultura e Biodiversidade – PPGAGRI, São Cristóvão, SE, Brasil; 3 Embrapa Tabuleiros Costeiros, Aracaju, SE, Brasil; 4 Embrapa Semiárido, Petrolina, PE, Brasil; 5 Universidade Federal de Sergipe – UFS, Departamento de Nutrição, São Cristóvão, SE, Brasil

**Keywords:** spermatozoa, fertility, ram, semen

## Abstract

The objective of this study was to evaluate the antioxidant properties of *Moringa oleifera* hay and the effects of its dietary inclusion on the reproductive potential of rams. Twenty-eight adult rams, with a mean age of two years and an average body weight of 69.36 ± 1.29 kg, were randomly allocated into four experimental groups (G) and subjected to isoproteic diets composed of 30% concentrate (corn, soybean meal, and urea), sorghum silage (SS), and MOH in different proportions: G0 (control: 0% MOH, 70% SS); G10 (10% MOH, 60% SS); G20 (20% MOH, 50% SS); G30 (30% MOH, 40% SS), along with *ad libitum* water. Semen collections were performed every 15 days for seminal and sperm evaluations. *M. oleifera* (MO) hay was analyzed for total phenolic compounds, flavonoid content, and in vitro antioxidant capacity and was compared with fresh MO. Total antioxidant capacity of seminal plasma was determined using the ferric reducing antioxidant power (FRAP) assay. MOH did not differ from MO in total phenolic content (33.38 vs. 33.64mg GA Eq/g), but its flavonoid content was higher (4.75 vs. 3.88 mg CAT Eq/g). DPPH (802.00 vs. 1840.38 µg/mL), ABTS (1091.78 vs. 2943.82 µg/mL), and FRAP (418.08 vs. 146.83 mg/mL) The results indicated greater antioxidant and reducing capacity in MO hay. No differences were observed among groups regarding total antioxidant capacity of seminal plasma or conventional seminal parameters (P> 0.05). However, regardless of the inclusion level, groups supplemented with MO hay showed a higher percentage of sperm plasma membrane integrity (P < 0.05) compared with the control group at all evaluation time points. It is concluded that the haymaking process enhances the antioxidant capacity of MO and that its inclusion in the diet of rams improves sperm plasma membrane integrity, positively reflecting semen quality. Thus, the use of MO hay may be considered a promising nutritional strategy to increase the reproductive potential of rams.

## Introduction

Spermatozoa are aerobic cells that depend on oxygen to maintain their metabolic functions and fertilizing capacity. However, excessive oxygen availability in the cellular environment may lead to overproduction of reactive oxygen species (ROS), which promote structural and functional damage to sperm cells. Under physiological conditions, a balance exists between ROS production and antioxidant defense mechanisms; however, disruption of this balance results in oxidative stress, compromising sperm viability (Andrade et al., 2010).

The plasma membrane (PM) of ram spermatozoa is highly sensitive to lipidic peroxidation due to its richness in polyunsaturated fatty acids (PUFAs) and cholesterol, which is important to maintain cell fluidity, as well as flexibility, acrosome reaction, and the storage of membrane-bound receptors (Bicudo et al., 2009). Since ruminants are not able to synthesize PUFAs, these fatty acids are found in their diet (Dolatpanah et al., 2008). The abundance of PUFAs in PM makes the spermatozoa more susceptible to oxidizing agents, such as free radicals and, thus, to lipidic peroxidation causing the loss of their functions leading them to death (Valença and Guerra, 2007).

The abundance of polyunsaturated fatty acids (PUFAs) in the plasma membrane renders spermatozoa highly susceptible to oxidative agents such as ROS, culminating in lipid peroxidation. These events promote structural and functional alterations in the sperm membrane, impairing fluidity, permeability, and cellular functionality, ultimately leading to loss of metabolic and fertilizing capacity and subsequent sperm cell death (Valença and Guerra, 2007).

Given this high sensitivity to oxidative stress, nutritional strategies have been investigated to minimize oxidative sperm damage, particularly through the inclusion of antioxidant-rich feed ingredients in diets of breeding rams (CBRA, 2013).

One feedstuff that has gained attention as a rich source of antioxidants is MO, a leguminous plant with high nutritional potential, containing significant amounts of PUFAs, as well as compounds such as oleic acid, glycosides, phenolic compounds including flavonoids and phenolic acids, organic acids, carotenoids, vitamin C, and other natural antioxidants (Sobral et al., 2020).

MO can be offered to ruminants with good acceptance either fresh or as silage or hay. The haymaking process provides additional advantages, including forage preservation, extended shelf life, utilization during periods of forage scarcity, and inclusion in concentrate diets as an alternative protein source capable of partially replacing conventional feed ingredients. Although several studies have demonstrated MO’s adaptability to climatic conditions, palatability, and nutritional value (Silva et al., 2019), information regarding the antioxidant properties of its hay and its potential effects on the reproductive potential of rams remains limited.

In this context, it is hypothesized that the haymaking process may maintain or even enhance the concentration of MO bioactive compounds, resulting in increased antioxidant activity. This activity may be associated with protection of the sperm membrane against oxidative stress-induced damage.

Considering the high susceptibility of ram spermatozoa to lipid peroxidation, antioxidants present in MO hay may reduce sperm lipid peroxidation, contributing to the maintenance of semen antioxidant defense balance. Therefore, the present study aimed to evaluate the antioxidant properties of MO hay compared with the fresh plant and to investigate the potential effects of its dietary inclusion on the reproductive potential of rams.

## Methods

The experimental procedures involving the animals were approved by the Animal Ethics Committee (AEC) at Brazilian Agricultural Research Corporation (EMBRAPA), under the protocol number 442022. The experiment was carried out at Embrapa Semi-arid Region, located in Nossa Senhora da Glória and Graccho Cardoso, Sergipe (10°13' South, 37°25’ West; altitude 291m) from December 7^th^, 2022 to February 8^th^, 2023. The laboratory analyses were performed at both Embrapa Coastal Tablelands and at the Federal University of Sergipe.

### Experimental animals

Rams were previously selected based on reproductive health evaluation, including general clinical examination, specific assessment of reproductive organs, and semen analysis, according to criteria established by the Brazilian College of Animal Reproduction (CBRA, 2013) and described by Martins et al. (2016) and Toker et al. (2016). Twenty-eight adult rams, with a mean age of two years and average body weight of 69.36 ± 1.29 kg, were sourced from the in situ Conservation Center of Santa Inês sheep at Embrapa Tabuleiros Costeiros. Animals were randomly assigned to four experimental groups according to the evaluated diets and housed in collective pens (seven rams per pen) measuring 2.8 × 3.6 m, providing 1.44 m^2^ per animal.

### Diets and feeding management

Experimental diets were formulated as isoproteic with 12% of raw protein and balanced to meet the demands of rams with an average daily weight gain of 100 g/animal/day (NRC, 2007). The diets were composed of sorghum silage (SS), corn concentrate, soybean meal, limestone, urea, and salt, besides different proportions of MOH (Table 1). The different diets determined the formation of experimental groups and varied according to the composition of the concentrate and the level of SS replacement by MOH as displayed in Table 2.

**Table 1:** Concentrate proportions of the different experimental groups.

**Experimental groups**				
**Food**	**G0 (%)**	**G10 (%)**	**G20 (%)**	**G30 (%)**
Soybean meal	30.0	20.3	11.4	2.0
Corn	66.5	76.2	85.4	95.0
Limestone	0.5	0.5	0.2	0.0
Salt	2.0	2.0	2,0	2.0
Urea	1.0	1.0	1.0	1.0
Total	100.0	100.0	100.0	100.0

Nutritional facts (g/kg): 200 calcium; 75 phosphorus; 90 sodium; 10 sulfur; 5 magnesium; 400 iron (mg/kg); 1,848 manganese; 3,060 zinc; 40 iodine; 20 cobalt; 24 selenium (IU/kg); 312,500 vitamin A; 50,000 vitamin D3; 437 vitamin E.

**Table 2:** Diets of experimental groups with different percentages of replacement of sorghum by MOH.

**Food**	**Experimental groups**			
	**G0 (%)**	**G10 (%)**	**G20 (%)**	**G30 (%)**
*Moringa oleifera* hay (MOH)	0	10	20	30
Sorghum silage (SS)	70	60	50	40
Concentrate (CC)^*^	30	30	30	30

* Composed of corn, soybean meal, limestone, urea, and salt.

The rams were fed for three days (D), as an adaptation period, followed by more 60 additional days (D1 to D60), considered as the effective experimental period for the analysis of the effect of the diets. All rams had *ad libitum* water access.

### Analysis of *Moringa oleifera* hay antioxidant properties

The antioxidant properties of MOH were analyzed from their extracts. These extracts were obtained by the mixture 100g of the MOH sample in 250mL of ethanol submitted to magnetic stirring for 24 hours. The supernatant content was then vacuum filtered and the ethanolic extracts were evaporated to dryness and concentrated. The extracts were stored in amber glass containers under refrigeration (2 °C). Stock solutions of the extracts were prepared at 1000μg/mL concentrations for the analyses to quantify the total phenolic and flavonoid compounds. Subsequently, aliquots of these solutions were used to obtain the concentrations of 10, 30, 100, and 300μg/mL, which were then used for the *in vitro* evaluation of the antioxidant capacity.

The bioactive analysis of MOH was based on the quantification of phenolic and flavonoid compounds and their extracts. The total phenolic compounds quantification was performed according to Swain and Hillis’s (1959) methodology, with minor modifications. Aliquots of 12.5μL of the extracts were diluted in a solution of 200μL of distilled water and 12.5 Folin-Ciocalteau reagent. After 3 minutes, 25μL of a saturated sodium carbonate solution were added. The mixture was kept undisturbed, protected from light, for one hour at room temperature. Then, the absorbances were measured using a plate reader at 720nm. The results were expressed as mg of gallic acid equivalents per g of extract, determined by a gallic acid standard curve (12.5 at 200μg/mL) under the same conditions.

^The flavonoid content was determined by the aluminum trichloride (AlCl^_3_^) method as^ described by Zhishen et al. (1999), with minor modifications. An aliquot of the extracts (25μL) was mixed with distilled water (100 μL), and later 7.5μL of NaNO_2_ (5%) were added. After 6 minutes, 7.5μL ^AlCl^_3_ (10%) were added to the mixture that was then kept undisturbed for more 6 minutes. After that, another solution of NaOH (4%, 100μL) and distilled water was added and kept undisturbed for 15 minutes. The intensity of the pink color was spectrophotometrically measured at 510nm. Catechin was used to construct the standard curve (0.125-2μg) and the results were expressed as mg of catechin equivalents per gram of extract.

The assays to measure the *in vitro* antioxidant capacity were performed in quintuplicate, evaluating the radical DPPH (2,2-diphenyl-1-picrylhydrazyl) scavenging capacity according to Brand-Williams et al. (1995) and the ABTS [2,2'-azino-bis (3-ethylbenzothiazoline-6-sulfonic acid] following Re et al. (1999); and the reducing capacity using the FRAP (Ferric Reducing Antioxidant Power) assay as described by Benzie and Strain (1996).

For the radical assay, 50µL of each extract were individually added to 150µL of a methanolic DPPH solution at 6x10-5 mol/L. The determinations included a negative control without antioxidants, in which methanol replaced the extracts. The DPPH radical reduction was measured at 517nm in a spectrophotometer right after being left undisturbed for 30 minutes. The decrease in absorbance (Abs) values of the extracts were correlated with the negative control and the percentage of the DPPH radical scavenging was determined by the following equation. The values were expressed in a half-maximal inhibitory concentration (IC50), corresponding to the quantity of samples needed to neutralize 50% of the DPPH solution.


$$\%\;Scavenging\;=\;100\;-\;\left[\left(Abs\;sample\;x\;100\right)/\;Abs\;negative\;control\right]$$ (1)

For the ABTS assay, a 30μL aliquot of the extracts was transferred to microplates containing 300μL of this radical representing 5mL of its stock solution at 7mM with 88μL of the potassium persulfate solution at 140mM. The microplate readings were taken using a spectrophotometer (734nm) after its calibration with ethanol as control and a 6-minute incubation period of the samples. The decrease in the absorbance values (Abs) of the samples were compared with the control, and, from that, the percentage of ABTS scavenging was established, with results expressed as IC50.


$$\%\;Scavenging\;=\;100\;-\;\left[\left(Abs\;sample\times 100\right)/\;Abs\;control\right]$$ (2)

For the evaluation of the reducing capacity, 9μL (concentration of 1000mg/mL) of each sample were transferred to a microplate, to which 27μL of distilled water and 270μL of the FRAP reagent were added. The FRAP reagent corresponds to 25ml of acetate buffer (0.3M), 2.5 mL of a TPTZ [2,4,6-Tris(2-pyridyl)-s-triazine] solution (10mM), and 2.5mL of an aqueous ferric chloride solution (20mM). The microplate was kept at 37 °C for 30 minutes and then evaluated using a spectrophotometer (595 nm), with the FRAP reagent serving calibration blank. Results were expressed in µM/mL of ferrous sulfate equivalents of extract. Results were expressed in µM/mL of ferrous sulfate equivalents of extract.

## Collection and semen analysis

Sperm analyses were performed using conventional methods routinely applied in semen evaluation of small ruminants, as recommended by the Brazilian College of Animal Reproduction. During the 60-day experimental period, semen was collected and analyzed every 15 days (D1, D15, D30, D45, and D60) to assess sperm parameters associated with reproductive potential.

Semen samples were obtained by electroejaculation using a TK 800® electroejaculator (Uberaba, Brazil). Animals were restrained in a guillotine-type chute and subjected to prior rectal ampulla cleaning. A lubricated probe with carboxymethylcellulose-based gel was inserted into the rectum, positioning the electrodes ventrally. Six low-voltage electrical stimuli (8 V) were applied, each lasting six seconds, at intervals of six to 10 seconds, as described by Damián and Ungerfeld (2011).

The semen collected was subjected to seminal and spermatic evaluations. The seminal evaluations comprised the volume (VL; mL), the spermatic concentration (CON; x10^6^sptz/mL), and the total number of spermatozoa (TNS; x10^9^sptz/mL) following adapted methodologies recommended by CBRA (2013). The CON was determined by adding an aliquot of semen to ultrapure water (Milli-Q®, Merck, Darmstadt, Germany) at a 1:400 ratio, followed by sperm counting using a Neubauer chamber under a light microscope (Nikon® E100, Japan) at 400x magnification. The TNS was calculated by multiplying the VL by the CON.

The spermatic evaluations included motility (MOT; %), vigor (VE; 0 a 5), spermatic morphology (%), plasma membrane integrity (%), and acrosome integrity (%). The MOT and VE were evaluated right after the collection by adding 10μL of semen to 500μL of X-Cell® (IMV, L’Aigle, France)® solution (IMV, L’Aigle, France) heated at 37 °C. A 10μL aliquot of the mixture was then transferred to a microscope slide, covered with a heated (37 °C) coverslip, and assessed using a light microscope (Nikon® E100, Japan).

The morphologic evaluation of the spermatozoa was performed through 10μL semen aliquot transfer to a 500μL phosphate saline buffer solution with 0.2% of glutaraldehyde heated at 37 °C. From this mixture, 10μL were extracted to have 200 spermatozoa analyzed using a phase-contrast light microscope (Nikon® E100, Japan) at 1000x magnification under immersion oil. The spermatozoa were classified according to major defects (MaD; %) and minor defects (MiD; %), and the sum of both was used to determine the percentage of total sperm defects (TD; %), which was used for comparison among experimental groups.

For plasma membrane integrity analysis, aliquots of semen stained with an eosin- nigrosin solution (1.1g eosin; 6.6g nigrosin; 0.5g sodium citrate; and 100mL of distilled water with pH between 6.8 and 7.0) at a ratio of 1:1 to 1:3 were used. The mixture was incubated for one minute at 36 °C for preparing a smear on a glass slide. Subsequently, the slides were dried at room temperature and kept for the counting of 200 spermatozoa in a light microscope (Nikon® E100, Japan) at 400x magnification in a bright field. Viable spermatozoa were consideered those with unstained heads (i.e., not pink colored with eosin) (Martins et al., 2016).

The acrosome integrity (AI; %) was assessed through a combination of Trypan Blue and Giemsa stains. Initially, 40µL of semen were mixed with 40µL of Trypan Blue solution 0.4% (Merck®, Brazil) in a 1.5mL tube and incubated for 15 minutes at 36 °C. Then, smears were prepared using 3µL to 4µL samples of this mixture, which were later air-dried and fixed in methanol for 5 minutes. Following fixation, the slides were allowed to dry at room temperature and subsequently stained for 8 hours in a Trypan Blue and Giemsa solution. After staining, the slides were rinsed with running water and allowed to air-dry naturally (Martins et al., 2016). A total of 200 spermatozoa were assessed in a light microscope (Nikon® E100, Japan) at 1000x magnification in a bright field, using immersion oil. The spermatozoa were classified according to the following patterns: blue head and dark pink acrosome (non-viable with intact acrosome); blue head with unstained acrosome (non-viable with reacted acrosome); light pink head with dark pink acrosome (viable with intact acrosome); light pink head with unstained acrosome (viable with reacted acrosome). Spermatozoa exhibiting a uniformly blue head were not included in the assessment of acrosome integrity.

For seminal plasma antioxidant capacity analysis, semen samples were centrifuged twice: the first one at 1000 x g for 10 minutes and the second at 4000 x g for 20 minutes. The supernatant plasma was collected and stored at -20 °C until analysis, following Glander et al. (2002) methodology. The seminal plasma samples were thawed and agitated on vortex mixer (Labnet®, USA) for 10 seconds. Then, a 9 μL aliquot of seminal plasma was transferred to microplates, and 27μL of distilled water and 270μL of FRAP reagent were added to each microplate. The mixture on the microplates was incubated at 37 °C for 30 minutes and absorbance was measured in a spectrophotometer (595 nm). The results were expressed in μM/mL of ferrous sulfate equivalents of seminal plasma.

### Statistical analysis

The data for bioactive compounds and for *in vitro* antioxidant capacity of the MOH and the MO are presented as mean ± standard deviation from three independent assays. Differences between MOH and MO samples were evaluated using Student’s t-test.

Data for the plasma membrane integrity and for the acrosome integrity, as well as for the total antioxidant capacity of the seminal plasma were subjected to a two way ANOVA, followed by Tukey’s post-hoc test.

The remaining semen evaluation data were analyzed using the SAS 9.1® software (SAS, 2012). As these data did not exhibit homoscedasticity and normal distribution, they were analyzed using non-parametric statistics. Specifically, multiple comparisons were performed using the Kruskal-Wallis test, and pairwise comparisons of means were conducted using Dunn's test. In all analyses, the significance level was set at 5% (P < 0.05).

## Results

### MOH antioxidant capacity

The total phenolic and flavonoid content and the antioxidant activity *in vitro* are presented in Table 3. When compared to MO, the MOH exhibited higher total flavonoid concentration, which explains its greater antioxidant activity, confirmed by the radical DPPH and ABTS scavenging assays, as well as by the FRAP reducing capacity. Significant differences in the total phenolic compound concentrations were not observed between MO and MOH.

**Table 3:** Bioactive compound content and antioxidant capacity in the ethanolic extract of MOH.

**Analysis**	**Results**	
	**Hay**	**MO In Natura**
Phenolic content (mg Eq AG/g)	33.38 ± 2.27a	33.64 ± 3.05a
Flavonoid content (mg Eq CAT/g)	4.75 ± 0.38a	3.88 ± 0.30b
DPPH IC50 (µg/mL)	802.00 ± 36.76a	1840.38 ± 160.45b
ABTS IC50 (µg/mL)	1091.78 ± 269.31a	2943.82 ± 460.20b
FRAP Ferrous sulfate equ (1000 mg/mL)	418.08 ± 18.17a	146.83 ± 16.89b

DPPH = 2,2-diphenyl-1-picrylhydrazyl; ABTS = 2,2'-azino-bis(3-ethylbenzothiazoline-6-sulfonic acid); FRAP = Ferric Reducing Antioxidant Power. IC50 = Half maximal inhibitory concentration; mg GAE/g = milligrams of gallic acid equivalents per gram; mg CE/g = milligrams of catechin equivalents per gram; data are presented as mean ± standard deviation. Different letters indicate significant differences (p < 0.05) between samples.

### Seminal and spermatic analysis

**Figure 1:**
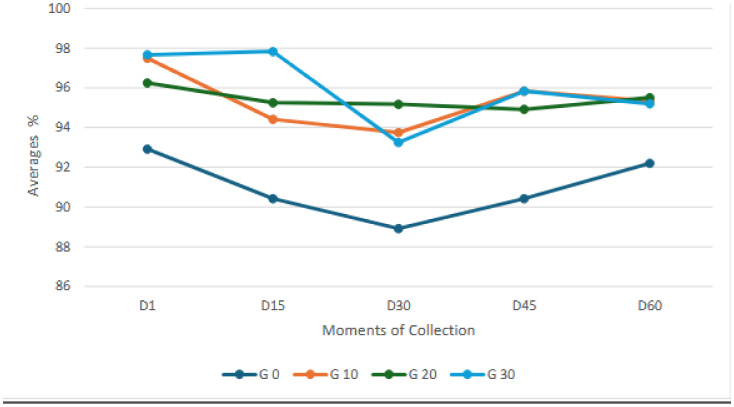
Analysis of the sperm plasma membrane integrity in the semen of rams fed with diets composed of different quantities of MOH.

The experimental groups did not differ significantly (p>0.05) in relation to the seminal analysis for volume, concentration, total number of spermatozoa, motility, vigor, morphology, and acrosome integrity (Table 4). Regardless of the concentration, the ram groups that received MOH in their diet presented higher (p<0.05) plasma membrane integrity compared to (GO) in each assessment (Figure 1). No significant difference was observed among the groups that received MOH in relation to this parameter (p>0.05).

**Table 4:** Assessment of the semen performed throughout the experiment in rams fed with different levels of MOH.

Collection	Analysis	Experimental Groups								
		G0		G10	G20	G30	Mean			S.E.
D1	VL (mL)	1.2		1.1	1.2	1.4	1.2			1.7
	CON (x10^6^ sptz/mL)	1.4		1.4	3.5	5.3	2.9			0.2
	TNS (x10^6^ sptz/mL)	1.6		1.5	4.2	7.3	0.3			1.5
	MOT (%)	66.7		70.0	75.0	71.7	70.8			0.7
	VE (1-5)	3.0		3.0	3.7	3.3	3.3			5.5
	TD (%)	4.0		4.0	1.7	4.8	3.6			0.1
	AI (%)	0.3		0.0	0.2	0.0	0.1			0.1
D15	VL (mL)	0.8		1.3	0.7	1.1	1.0			0.1
	CON (x10^6^ sptz/mL)	2.4		1.3	2.2	3.6	2.4			0.5
	TNS (x10^6^ sptz/mL)	1.9		1.6	1.6	4.0	2.3			0.1
	MOT (%)	71.7		73.3	71.7	71.7	72.1			0.4
	VE (1-5)	3.2		3.2	3.2	3.2	3.2			0.0
	TD (%)	9.6		9.6	2.3	4.0	6.4			1.9
	IA (%)		0.0	0.0	1.0	0.8		0.5	0.3	
D30	VL (mL)		1.1	1.2	1.0	1.2		1.1	0.0	
	CON (x10^6^ sptz/mL)		3.2	1.2	3.1	3.2		2.7	0.5	
	TNS (x10^6^ sptz/mL)		3.5	1.5	3.1	3.9		3.0	0.0	
	MOT (%)		70.0	73.3	73.3	68.3		71.3	1.3	
	VE (1-5)		3.2	3.2	3.3	3.2		3.2	0.0	
	TD (%)		6.6	6.6	17.3	10.9		10.3	2.5	
	AI (%)		32.8	57.2	47.5	65.5		50.7	7.0	
D45	VL (mL)		1.1	1.0	0.8	1.3		1.1	0.1	
	CON (x10^6^ sptz/mL)		2.8	2.5	1.6	3.7		2.7	0.4	
	TNS (x10^6^ sptz/mL)		3.1	2.5	1.3	4.8		2.8	0.0	
	MOT (%)		68.3	76.7	73.3	75.0		73.3	1.8	
	VE (1-5)		3.2	3.2	3.3	3.3		3.3	0.1	
	TD (%)		11.4	11.4	8.8	7.0		9.7	1.1	
	AI (%)		0.0	0.0	6.3	0.3		1.7	1.6	
D60	VL (mL)		1.1	1.2	1.0	1.1		1.1	0.0	
	CON (x10^6^ sptz/mL)		2.7	1.5	3.1	1.4		2.2	0.4	
	TNS (x10^6^ sptz/mL)		3.0	1.7	3.2	1.5		2.4	0.0	
	MOT (%)		71.7	75.0	76.7	78.3		75.4	1.4	
	VE (1-5)		3.3	3.3	3.7	3.8		3.5	0.1	
	TD (%)		4.5	4.5	2.1	1.3		3.1	0.8	
	AI (%)		3.7	37.0	16.5	0.3		14.4	8.3	

G0 = 0% MO; G10 = 10% MO; G20 = 20% MO; G30 = 30% MO; D1 = first collection on the first effective administration day after three days of diet adaptation; D15 = Second collection after 15 days of the effective diet administration period; D30 = Third collection after 30 days of the effective diet administration period; D45 = Fourth collection after 30 days of the effective diet administration period; D60 = Fifth collection after 60 days of the effective diet administration period; VL = semen volume; TNS = total number of spermatozoa; CON = spermatic concentration; MOT = motility; VE = vigor; TD = total sperm defects; AI = acrosome integrity; Sptz = spermatozoa; S.E. = Standard Error.

Differences observed in plasma membrane integrity were consistent throughout the entire experimental period, as shown in Figure 1, regardless of evaluation time. No significant difference was observed among groups receiving MO hay for this parameter (P > 0.05).

**Figure 2:**
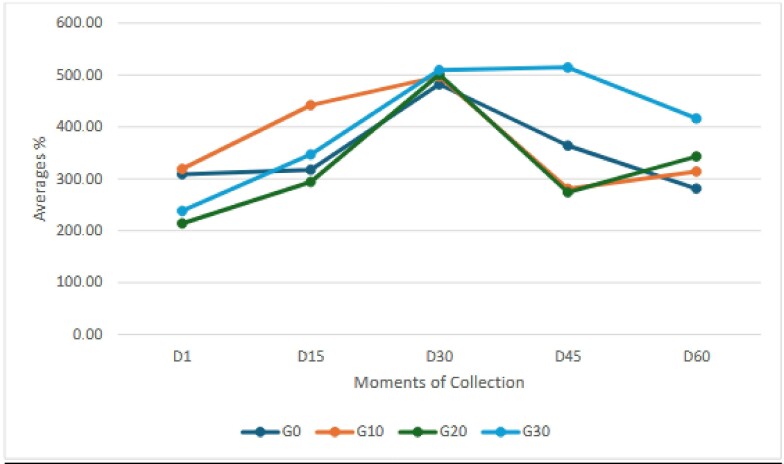
Total antioxidant capacity (FRAP) of the seminal plasma of rams fed diets containing different levels of MOH.

Even though, the *in vitro* methods have shown that MOH has high antioxidant capacity, the diets containing this element did not increase the total antioxidant capacity of the seminal plasma (p>0.05), according to the FRAP method (Figure 2).

## Discussion

In this study, a high flavonoid content was observed in MO hay, which at least partially explains its marked antioxidant capacity. The haymaking process proved efficient in preserving and, in some cases, enhancing the content of bioactive compounds, resulting in greater antioxidant activity compared with fresh MO. Similar findings were reported by Sobral et al. (2020) and Ramírez-Bautista et al. (2020), who demonstrated that processing methods capable of reducing moisture content may increase the relative concentration of phenolic metabolites and, consequently, the antioxidant activity of forages.

Dietary inclusion of MO hay promoted a consistent increase in sperm plasma membrane integrity, a structure essential for maintaining cell viability and sperm functionality. This finding is particularly relevant, as plasma membrane integrity is directly associated with protection against oxidative damage and preservation of semen fertilizing potential (Wafa et al., 2017). Thus, even in the absence of significant changes in other classical seminal parameters, improvement in this cellular attribute indicates a beneficial effect of MO hay on sperm quality.

Seminal parameters related to overall semen quality, such as volume, sperm concentration, and total sperm number, remained within normal ranges established by CBRA (2013) in all experimental groups, including those receiving MO hay. Observed semen volumes were within the normal range for sheep (0.5 to 2.0 mL), as described by Maia et al. (2011) and Ramírez-Bautista et al. (2020), varying according to factors such as collection method and frequency of use of breeding males. Similarly, Syarifuddin et al. (2021) reported no changes in semen volume of goats fed diets containing MO leaves.

Sperm motility showed mean values above 70% in all experimental groups, corroborating findings reported by Shokry et al. (2020). However, these values were lower than those considered ideal for fresh ovine semen according to CBRA (2013). This result may be attributed to a combination of factors, including lack of prior genetic selection of the Santa Inês rams used, climatic conditions during the experimental period characterized by high temperatures in the semi-arid region of northeastern Brazil, and exposure to stressors such as transportation, adaptation to the experimental environment and diets. Additionally, the electroejaculation method, known to present greater variability, may have contributed to the reduction in these values. Nevertheless, the observed percentages can be considered reproductively satisfactory.

Previous studies have reported positive effects of MO supplementation on seminal and sperm characteristics, partially differing from the findings of the present study. Shokry et al. (2020) observed increased semen volume and sperm concentration in sheep supplemented with alcoholic extract of MO leaves, suggesting that the form of administration and bioavailability of bioactive compounds may influence reproductive responses.

## Conclusion

This study demonstrates the effects of including *Moringa oleifera* hay in the diet of rams on reproductive parameters. Improvements in plasma membrane integrity and in the antioxidant capacity of seminal plasma indicate a potential positive impact on reproductive efficiency. Dietary interventions based on natural sources may represent promising strategies for more sustainable livestock production systems with lower environmental impact.

## Data Availability

Research data is available in the body of the article.
